# The Smethurst Case

**Published:** 1859-12

**Authors:** 


### THE SMETHURST CASE.

The necessary limitation of space prevents us from noticing in the briefest terms upwards of forty other cases of forensic interest which have appeared since our last report in the British and Foreign Journals. We must, however, for the sake of our Continental readers, say a word respecting the famous Smethurst case, which in this country has for some months been the leading medical topic. This case was one in which poison was suspected; but as there was no evidence of poisoning as the cause of death, save in the imagination of men who have gifts in that direction, we could not logically put the case in our toxicological section. The essence of the case is that a woman, forty-three years of age, and having premonitory indications of abdominal disease, became pregnant. That upon the pregnancy there resulted vomiting, an aggravation of the intestinal disturbance and dysentery. In great measure from starvation (owing to the inability of the stomach to retain food), to which must be added the exhaustion produced by the dysenteric disease, death was the result. *Facilis descensus averni:* the medical philosophers who attended the case, not appreciating the meaning of the previous history of the woman, ignoring altogether the fact of pregnancy, and mistaking the symptoms which peculiarly mark starvation, came to the hypothesis that the case was one of slow irritant poisoning. Nevertheless, they took no pains for many days—not indeed until death was at the door—either to remove the poisoner, to look for poison, or to give an antidote. On the contrary, they prescribed for the case as one of ordinary disease almost to the end. When, granting that the case had been a case of poison, it was too late to do anything, the supposed poisoner was arrested, the antidote was supplied, and the poison was searched for. Suffice it to add, that no poison was found in the possession of the prisoner; none *satisfactorily* in the body of the patient after her death; that the post-mortem appearances were admitted to be dysentery; and that the fact of the existence of pregnancy was revealed by the knife. In spite of all, the vague hypothesis was adhered to. A man was tried for his life, and a jury, overpowered by the dogmatic, and, we may add, stereotyped statement of the scientific witnesses for the prosecution; to wit, that death could only be attributed to irritant poisoning—condemned the prisoner to the scaffold. Since the condemnation the country has risen up against it, and the Secretary of State for the Home Department, than whom a more logical mind is not in this realm, has granted a reprieve from death. The nation is thus happily relieved from a national sin, and English science rescued from indelible disgrace.—*Med.-Chir. Rev.*

### THE CASE OF THOMAS SMETHURST.

The reprieve of Smethurst may be regarded as the verdict of the country. It is a remarkable example of the value of public discussion. It is, above all, a signal proof of the pure love of justice that stamps the national character and institutions of England. It would be difficult to point to any convict who has excited so little personal sympathy as Smethurst. His previous career, and the circumstances of his connection with Miss Bankes, are so marked by low selfishness that no feeling akin to tenderness or pity can be entertained towards him. Not even the plea of passion too wild to bear the restraints of law or religion can be urged in extenuation of his conduct. Sordid, calculating venality appears through all. Notwithstanding the absence of any personal quality or collateral circumstance to stimulate the movement in his favor, he has been snatched from the gallows by the almost unanimous decision of his countrymen, who, reviewing the case upon its abstract merits, find that the heavy charge upon which he was found guilty by a jury is not substantiated by the evidence. Casting aside all feeling for or against the prisoner, unswayed by the circumstances that seem to establish a motive for the crime and to justify a strong suspicion of foul play, the country, acting as a court of appeal, has deliberately analyzed the evidence, and, finding it defective in the essential points, has cancelled the verdict of the jury. It may be doubted whether in any other country a revision of the sentence of a legally-constituted tribunal would be conducted by the press and by the people in a temper equally free from excitement, and in a spirit so purely vindicatory of public justice. The history of such a case as this may well be referred to as an answer to those foreign jurists who reproach us with the want of a court of appeal in criminal cases. With a free press, with the habit of judicial investigation, and the simple love of justice that animates every citizen amongst us with the resolve to see right dealt out to every one, we stand, perhaps, in less need of a complicated ascending scale of criminal courts than is experienced in countries where the tribunals receive little or no aid from external discussion. In this case it may be said that, through the machinery of the press, not Smethurst, but the Law, has enjoyed the benefit of the experience of the entire body of the medical profession, in the elucidation of the obscure scientific questions upon which the verdict turned. The evidence elicited before the jury has not only called forth fresh testimony, but has naturally challenged ths criticisms of the profession. The result is a partial, if not wholly satisfactory, vindication of justice. The spirit of English law yet calls for something more than a reprieve. If it be admitted—and the reprieve actually admits so much—that Smethurst is not proved to be guilty of the crime of murder for which he was arraigned, he is logically and justly entitled to an absolute discharge. If he is still held a prisoner, it must be either for the crime of which he is admitted to be innocent, or for some other crime for which he has not been put upon his trial. But we cannot in this place pursue the inconsistencies of our criminal law. We may leave them with the less regret, under the conviction that the resulting evils are mitigated by the wholesome action of public opinion. Although Thomas Smethurst, now declared not proved guilty of murder, can only be released by the granting of her Majesty's pardon for that offence, the gross inconsistency that strikes the moral sense can work but little harm, in the face of that universally-operating public opinion which sees through the fiction, and reduces it to a technical form.

We reserve for our next publication a critical epitome of this now celebrated cause, the special object of which will be to bring out its medico-legal bearings. Our present purpose is simply to advert, in a general manner, to the public interest of the case as illustrating the advantages accruing from a free discussion of the evidence; and the position more especially, of Medicine as an ally of the Law. To some, perhaps to many, it may appear that Medicine in this case has been at fault; that since it was made to support a charge of the heaviest degree of criminality, which upon more rigorous examination could not be sustained, scientific testimony has lost its claim to some of that confidence which it challenges before the world. It is no part of our duty to defend indiscriminately every medical witness. The profession are only concerned for the just credit of Medicine. We may observe, that if Medicine may seem to have sustained some little hurt in this trial, it is not because science was at fault, but because she was unfortunate in the particular individuals who chanced to be her exponents. It so happened that the witnesses for the prosecution failed to grasp all the scientific elements in the question before them, and consequently, to appreciate at their just value the facts which fixed their attention. But their omissions have been amply supplied by the correcting experience and more deliberative judgment of the witnesses for the defence, and still more by the re-examination of the evidence by the general body of the profession. In this way Medicine has in the end fully asserted her competency and usefulness. By common consent, the public has admitted the insufficiency of the moral and general evidence to justify conviction for the crime of murder. The moral evidence at most raises a presumptive motive—suggests that Smethurst had an interest in destroying Miss Bankes. Of direct evidence, such as the possession of poison by the prisoner, mixture of poison with the food or medicine, there is none. The whole case rested, in the first place, upon the scientific evidence. That must first be proved which Medicine only is competent to prove—namely, that the deceased died by poison; then the moral and general evidence which suggests that the prisoner was the most likely person to administer the poison, comes to bear. But unless the first—the strictly medical point—be decided in the affirmative, the second point has no existence—the general evidence goes for nothing. Now if, trusting too implicitly to the partly erroneous and generally fallacious testimony of the scientific witnesses for the prosecution, the jury came to the conclusion that Miss Bankes did die from the effects of poison, the appeal after the trial to the wider experience and unprejudiced judgment of the profession completely proved the fallacy of the first conclusion, and established the perfect competency of Medicine to pronounce a satisfactory decision upon the case as presented to the public. That decision has, in fact, been accepted by the public; and its ratification by the Home Secretary is the final acknowledgment of the services that science has in this case rendered to the administration of justice.—*Lancet.*

